# Poly(4‐Vinylpyridine)‐Based Cubosomes: Synthesis, Assembly, and Loading Capabilities

**DOI:** 10.1002/smsc.202400274

**Published:** 2024-10-17

**Authors:** Marcel Schumacher, Nadine Tänzer, Marius G. Braun, Manuel Trömer, Giada Quintieri, Mahima Goel, Markus Heidelmann, André H. Gröschel

**Affiliations:** ^1^ Institute of Physical Chemistry and Center for Soft Nanoscience (SoN) University of Münster Busso‐Peus‐Str. 10 Münster 48149 Germany; ^2^ International Graduate School BACCARA University of Münster Münster 48149 Germany; ^3^ Polymer Materials for Energy Storage (PES), Bavarian Center for Battery Technology (BayBatt) and Bavarian Polymer Institute (BPI) University of Bayreuth Universitätsstr. 30 Bayreuth 95448 Germany; ^4^ Interdisciplinary Center for Analytics on the Nanoscale (ICAN) University of Duisburg‐Essen Duisburg 47057 Germany

**Keywords:** block copolymers, hybrid materials, inverse morphologies, mesoporous microparticles, self‐assemblies

## Abstract

Polymer cubosomes (PCs) are 3D porous microparticles with high surface area that have great potential for applications that require a large interfacial area including catalysis, drug delivery, and energy storage. Most reported PCs are based on chemically inert block copolymers (BCPs) with limited intrinsic functionality, which is why they have been mainly used as templating material. Herein, the synthesis, self‐assembly, and loading of poly(4‐vinylpyridine) (P4VP)‐based PCs are reported. The pyridinic moieties are located inside the PC wall and are well‐known functional groups for coordination, cross‐linking, and pH response, which is demonstrated on platinum coordination and pH‐dependent dye release.

## Graphical abstract

Polymer cubosomes (PCs) were self‐assembled from poly(ethylene oxide)‐*b*‐poly(4‐vinylpyridine) (PEO‐*b*‐P4VP) block copolymers (BCPs) with high P4VP block lengths of over 400 units in mixtures of dimethylformamide (DMF) and sodium hydrogen carbonate (NaHCO_3_). The PC morphology was verified by scanning and transmission electron microscopy, while the functionality of the 4‐vinylpyridine units was exemplified by loading platinum complexes and organic dyes.

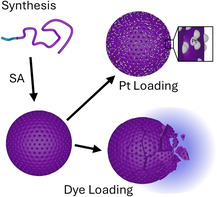

## 1 Introduction

Polymer cubosomes (PCs) are a recent type of block copolymer (BCP) nanostructure which can be directly obtained through self‐assembly of asymmetric BCPs in solution.^[^ ^1^, ^2^, ^3^, ^4^ ^]^ PCs are mesoporous microparticles with a bicontinuous inner channel system of long‐range order that penetrates the entire structure. One of the pore channels is open to the surroundings and can be utilized for loading, release, and templating.^[^ ^5^, ^6^ ^]^ The accompanied large interfacial area makes PCs interesting for a variety of applications including catalysis,^[^ ^7^, ^8^, ^9^ ^]^ drug delivery,^[^ ^10^, ^11^ ^]^ capture and release,^[^ ^12^, ^13^ ^]^ energy storage,^[^ ^14^, ^15^ ^]^ and several others.^[^ ^16^ ^]^

PCs are inspired by lipid cubosomes, which are formed by monoolein or its derivatives that have a large hydrophobic volume fraction as compared to their small hydrophilic head group.^[^ ^17^, ^18^, ^19^ ^]^ The resulting packing parameter, *p*, is greater than one driving the assembly into open porous micelles termed cubosomes and hexosomes.^[^ ^20^, ^21^, ^22^, ^23^, ^24^ ^]^ While lipid cubosomes are liquid, PCs are made from amphiphilic BCPs with high intrinsic viscosity, which makes them mechanically more robust.^[^ ^25^, ^26^ ^]^ Through BCP design, PCs are tunable in particle dimension, pore diameter, lattice parameter, and in principle, also regarding surface and wall chemistry. However, with only few exceptions,^[^ ^27^, ^28^, ^29^, ^30^, ^31^ ^]^ most PCs consist of polystyrene (PS), whose good mechanical stability enabled their use as templates, but otherwise lack advanced functionalities (e.g. responsiveness, coordination sites, degradability, etc.).

**Scheme 1:**
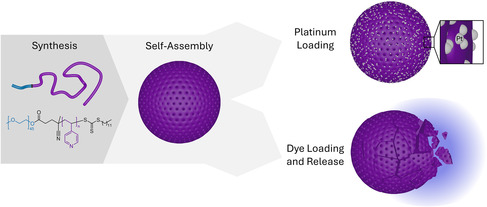
PEO‐*b*‐P4VP block copolymer, cubosome formation, loading, and release.

In this work, we synthesize functional PCs based on poly(ethylene oxide)‐*b*‐poly(4‐vinylpyridine) (PEO‐*b*‐P4VP), where the P4VP block forms the cubosome wall. The introduction of the pyridinic groups opens up versatile possibilities for modification, as the nitrogen atom can be chemically addressed in various ways.^[^ ^32^, ^33^ ^]^ For instance, P4VP can be loaded with a wide range of inorganic materials,^[^ ^34^ ^]^ it can undergo hydrogen bonding with various organic substances (e.g., phenolic hydroxyls),^[^ ^35^ ^]^ and it is pH sensitive, i.e., P4VP is hydrophobic under basic conditions and fully water‐soluble under acidic conditions.^[^ ^36^, ^37^, ^38^ ^]^ We here exemplified the modification potential by selectively loading platinum into the P4VP wall, as well as loading organic model dyes that can be release depending on the pH (**Scheme** **1**).

## 2 Results and Discussion

### 2.1 Polymer Synthesis

**Figure 1:**
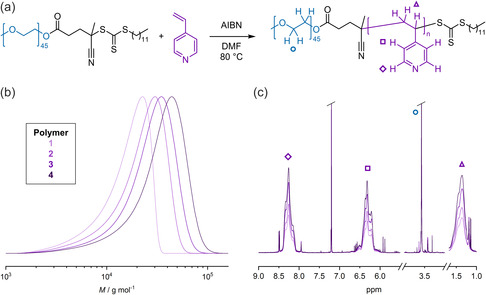
Synthesis and characterization of PEO‐*b*‐P4VP BCPs. a) Reaction scheme of the RAFT polymerization. b) Molecular weight distributions of BCP 1–4 measured with SEC. c) NMR spectra showing the increase of P4VP specific signals relative to PEO at 3.58 ppm.

The conditions for radical addition‐fragmentation chain‐transfer (RAFT) polymerization are briefly outlined here with BCP specifics summarized in **Table** **1**. More details can be found in Section 4. First, the PEO–chain‐transfer agent (CTA) was synthesized *via* Steglich esterification of the RAFT agent, 4‐cyano‐4‐(((dodecylthio)carbonothioyl)thio)pentanoic acid to PEO methyl ether with an average molar mass of 2000 g mol^−1^ using *N*,*N*′‐dicyclohexylcarbodiimide (DCC) and 4‐dimethylaminopyridine (DMAP) in dichloromethane (DCM). The reaction proceeded with almost quantitative conversion of 99.5% (nuclear magnetic resonance spectroscopy [NMR]) and resulted in a PEO–CTA with a low dispersity of 1.02 according to size‐exclusion chromatography (SEC). Subsequently, the PEO–CTA was used for the RAFT polymerization of 4‐vinylpyridine (4VP) in dimethylformamide (DMF) using azobisisobutyronitrile (AIBN) as radical initiator (**Figure** **1**). BCPs with different length of the P4VP block and sufficiently low dispersities^[^ ^39^ ^]^ could be obtained by stopping the reaction at different times (Figure 1b). According to NMR, the molar mass of the P4VP block ranged from 22.0 to 47.1 kg mol^−1^, which is equivalent to P4VP weight fractions (*f*_P4VP_) of 90.9% to 95.8% (Figure 1c and Figure S1, Supporting Information). The *f*_P4VP_ covers a broad range for which PCs have already been found for other BCPs.^[^ ^16^ ^]^

**Table 1:** Polymer specifics of the PEO_45_‐*b*‐P4VP_n_ BCPs synthesized for this work.

BCP	DP 4VPa) [units]	*f*_P4VP_ b) [%]	*M*_n,NMR_ c) [kg mol^−1^]	*M*_n,SEC_ d) [kg mol^−1^]	*Đ* d)
1	187	90.9	22.0	12.6	1.36
2	262	93.3	29.9	17.1	1.44
3	316	94.4	35.6	21.0	1.44
4	426	95.8	47.1	25.7	1.50

a) Degree of polymerization for the 4VP units of the BCPs determined by NMR.

b) Weight fraction of P4VP determined by NMR.

c) Number average molar mass determined by a combination of SEC and NMR.

d) Number average molar mass and dispersity determined by SEC in DMF (using PS standard).

### 2.2 Formation of PEO‐*b*‐P4VP Cubosomes and Characterization

Self‐assembly was accomplished *via* nanoprecipitation, i.e., a selective solvent was added to the BCP solution through a syringe pump to reach a final solvent composition at which self‐assembly is completed. This system deviates from previously reported PCs, because the hydrophobic block is comparably polar and can be protonated, which needs to be taken into account when adapting the self‐assembly process.^[^ ^16^ ^]^ Therefore, we first screened various conditions including 1) different common solvents, 2) BCP concentration, 3‐5) type, addition volume, and addition rate of selective solvent. All self‐assembly experiments were carried out using DMF as starting solvent, as other protic, aprotic polar, aqueous acidic solvents were tested unsuccessfully (Figure S2, Supporting Information). Moreover, PEO‐*b*‐P4VP BCPs proved insoluble in nonpolar solvents like dioxane, which is typically used as starting solvent for cubosome formation from other BCPs.^[^ ^40^ ^]^

**Figure 2:**
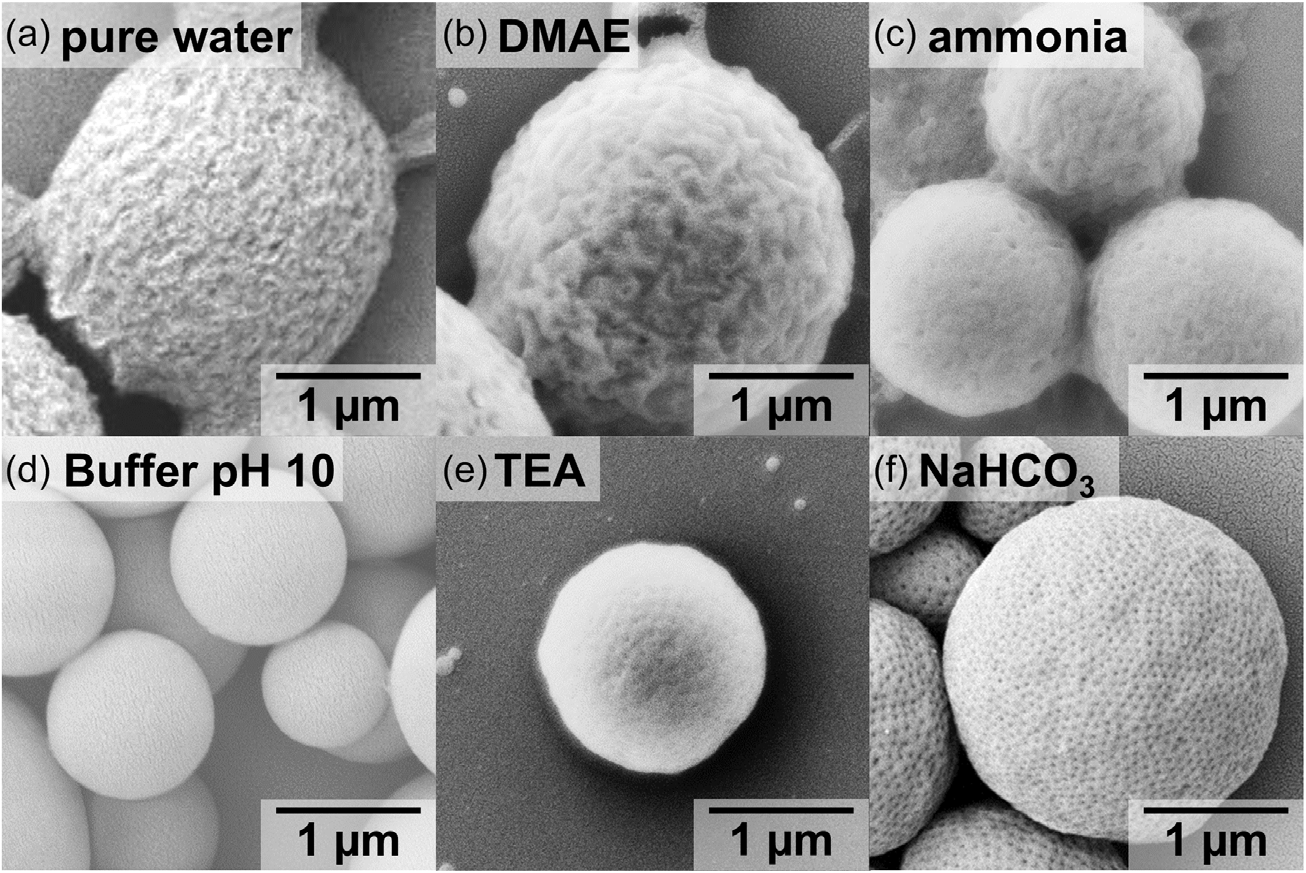
SEM images of the self‐assembly of 10 mg BCP 3 (*f*_P4VP_ = 94.4%) in 1 mL DMF with 2 mL of selective aqueous solvents: a) pure water, b) dimethylaminoethanol (DMAE), c) ammonia, d) borate buffer pH 10, e) triethylamine (TEA), and f) sodium hydrogen carbonate NaHCO_3_.

To find the best selective solvent (**Figure** **2**), we performed a series of self‐assembly experiment using 10 mg of BCP3 (*f*_P4VP_ = 94.4 %) in 1 mL DMF as the starting solution followed by the addition of a total volume of 2 mL selective solvent with an addition rate of 0.5 mL h^−1^. As selective solvents we chose either pure Milli‐Q water or an aqueous solution of dimethylaminoethanol (DMAE), ammonia, borate buffer pH 10, triethylamine (TEA), or sodium hydrogen carbonate (NaHCO_3_). Finally, the samples were dialyzed against the respective selective solvent to remove remaining DMF. Pure water would usually be the obvious choice for self‐assembly of amphiphilic PEO BCPs, but as can be seen from the scanning electron microscopy (SEM) image in Figure 2a, the dropwise addition of Milli‐Q water did not lead to the expected PCs but to spongelike structures. Unlike most hydrophobic blocks used for PC formation (e.g., PS), P4VP is a rather polar block and the aromatic nitrogen is sufficiently protonated to dissolve the polymer at pH ≤ 5 (p*K*_a_ ≈ 4).^[^ ^41^, ^42^ ^]^ Therefore, Milli‐Q water with a pH ≈ 5.5 was not feasible, as partial protonation could interfere with the assembly process. We thus changed the selective solvent to basic aqueous solutions containing DMAE and ammonia (pH 10), which however likewise resulted in spongelike structures (Figure 2b,c). DMAE is a solvent for P4VP, and even smaller amounts could therefore plasticize or even dissolve the characteristic cubosome structure during sample preparation for analysis (DMAE evaporates slower than water). Ammonia in contrast is known for its ability to cleave the dithiocarbonate group of the RAFT agent leading to a thiol (‐SH) group at the end of the polymer chain instead of the dodecyl trithiocarbonate.^[^ ^43^ ^]^ In the presence of oxygen, ‐SH groups could link two AB diblock chains into an ABA triblock copolymer via disulfide bridges, which would also interfere with assembly.^[^ ^44^ ^]^ In case of the borate buffer with a pH of 10, merely spherical particles could be detected with a closed surface probably caused by the high salinity (Figure 2d). Finally, TEA and NaHCO_3_ resulted in spherical PEO‐*b*‐P4VP microparticles with clearly visible porosity that is typical for PCs (Figure 2e,f). Although TEA has the advantage of evaporating during drying, it is classified as toxic^[^ ^45^ ^]^ and also resulted in slightly less defined PCs as compared to NaHCO_3_. The P4VP cubosomes are not only stable in basic water, but once formed, the can also be transferred to nonpolar organic solvents like toluene^[^ ^46^ ^]^ or ether,^[^ ^47^ ^]^ as P4VP is insoluble in these and other less polar solvents.^[^ ^48^ ^]^

Apart from the type of selective solvent, we investigated the effect of BCP concentration and tested 0.1 and 25 mg mL^−1^, for which the conditions discussed earlier were applied, i.e., 2 mL of 10 mM NaHCO_3_ were added to 1 mL BCP3 in DMF with a rate of 0.5 mL h^−1^. Surprisingly, both lower and higher concentration resulted in less defined structures likely due to changes in aggregation kinetics. Lower concentrations led to small particles with a closed surface, while higher concentrations led to a more random pore spacing and a partially closed surface. During addition of selective solvent, the homogeneous BCP solution transitioned through a state of liquid–liquid phase separation (LLPS) into BCP‐rich droplets that refined into the PC structure during further addition of selective solvent.^[^ ^49^, ^50^ ^]^ If the BCP concentration was too high however, the structural evolution within the highly concentrated droplets experienced different assembly kinetics (higher viscosity, kinetically trapped states). At low concentration, the BCP‐rich droplets would shrink until a critical concentration for aggregation was reached, which occurred at a higher fraction of selective solvent. In either case, deviations from the ideal PC morphology are to be expected. To test the effect of added volume of selective solvent, we added varying volumes of 10 mM NaHCO_3_ solution (Figure S2, Supporting Information), while keeping the other conditions constant (1 mL BCP3/DMF with 10 mg mL^−1^, addition rate of 0.5 mL h^−1^). The addition of 0.5 mL NaHCO_3_ solution did not lead to LLPS or particle formation as the solution remained clear without any noticeable turbidity. After 1.0 mL, the solution became slightly turbid and after 2 mL the solution turned milky. Since a milky solution is typically a sign for light scattering on particles >500 nm, we assumed that microparticle formation had occurred. We analyzed the morphologies at 0.5 mL and 1.0 mL by rapid quenching of the BCP solution in excess NaHCO_3_ solution and found closed spherical microparticles with some surface porosity, but of overall lower quality than with 2.0 mL added. The BCPs are presumably still located in solution or in BCP‐rich droplets, which were quenched by dialysis against NaHCO_3_. Thereby, the solvent rapidly diffused out of the BCP‐rich droplets into the continuous phase and the BCPs did not have a sufficient time to properly arrange themselves into the energetic minimum. Therefore, we concluded that the PC morphology was not sufficiently developed at such low selective solvent content. We argue that a comparably large amount of selective solvent is needed, because P4VP is rather polar for which water is not as strong a selective solvent as for other BCPs.

Finally, addition rates of selective solvent were changed to 1 and 2 mL h^−1^ (Figure S4, Supporting Information). We found that slower addition rates (0.5 mL h^−1^) are beneficial and produce more defined structures than medium addition rates (1 mL h^−1^) or fast addition rates (2 mL h^−1^). In the latter case, we even found partially closed pores on the surface of the particles suggesting that slower addition rates give BCPs more time to arrange into the defined cubosome lattice, while fast rates quench the process too rapidly.

**Figure 3:**
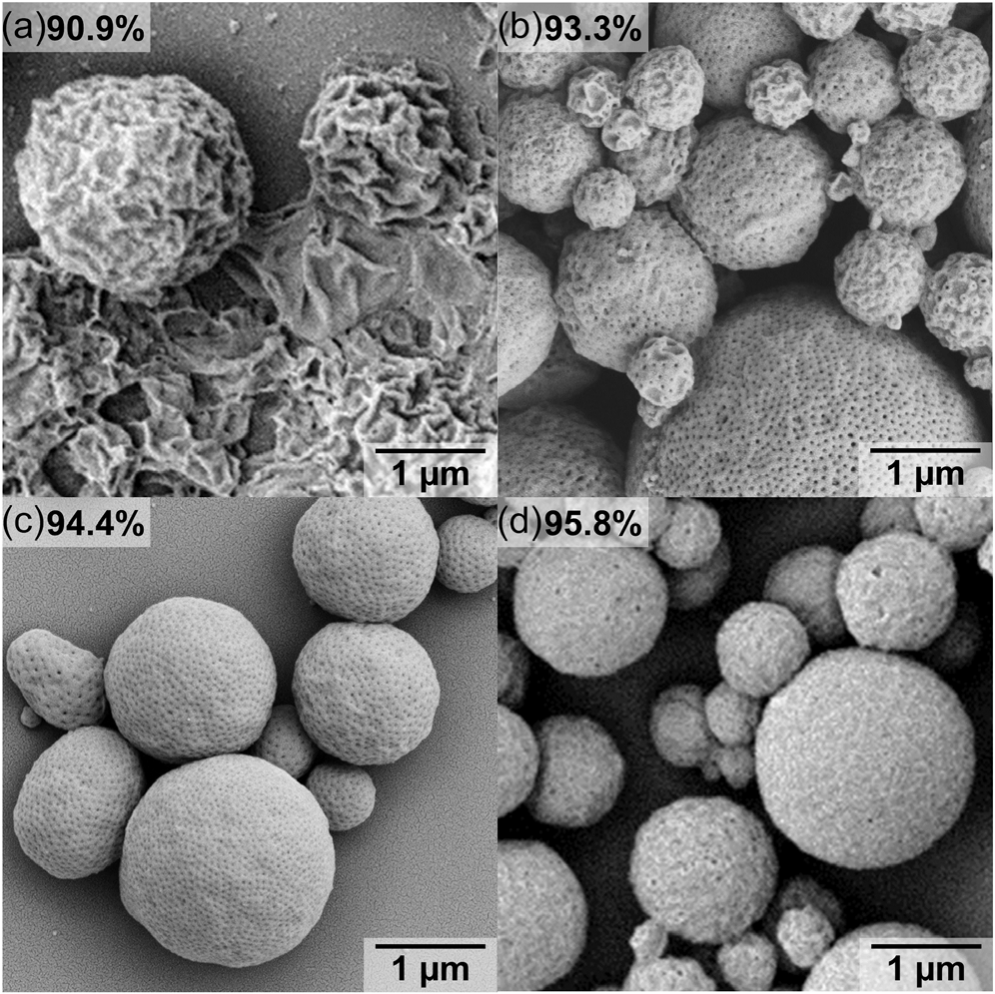
SEM analysis of PEO‐*b*‐P4VP morphologies with different *f*_P4VP_: a) 90.9 wt%, b) 93.4 wt%, c) 94.4 wt%, and d) 95.8 wt%. Conditions: 2 mL 10 mM NaHCO_3_ solution added to 1 mL BCP3/DMF with 10 mg mL^−1^ at 0.5 mL h^−1^.

After having identified the best preparation conditions (10 mg of BCP3 in 1 mL DMF, adding 2 mL of 10 mM NaHCO_3_ in 4 h), we subjected all BCPs 1–4 to the same assembly conditions to analyze the effect of *f*_P4VP_ on morphology (**Figure** **3**). With increasing *f*_P4VP_, the packing parameter is increasing and inducing a transition from polymersomes to PCs and, potentially even polymer hexosomes.^[^ ^51^, ^52^, ^53^ ^]^ With a hydrophobic ratio of about 90.9% (Figure 3a), only spongelike microparticles are obtained, as *p* is in between the region of polymersomes and PCs. These particles are porous throughout but lack the homogeneity of the pore diameter distribution associated with PCs. With 93.3% (Figure 3b), a mixture of spongelike microparticles and PCs can be found; they have a mean pore diameter of 23 ± 7 nm and a mean pore spacing of 106 ± 18 nm, which is already comparable to PCs. With at a slightly higher *f*_P4VP_ of 94.4% (Figure 3c), PCs became the dominant morphology with the highest quality, which is quantified by the pore diameter and its narrow distribution of 19 ± 3 nm and pore spacing of 80 ± 4 nm. At even higher hydrophobic ratios of 95.8%, the surface of obtained microparticles is still porous, but not as ordered as before (Figure 3d). The average pore diameter of 45 ± 23 nm is comparably large and broad, just like the pore spacing of 174 ± 73 nm. The packing parameter could be too large and the hydrophobic block too long thereby exceeding the range for PC formation.^[^ ^4^, ^39^ ^]^

**Figure 4:**
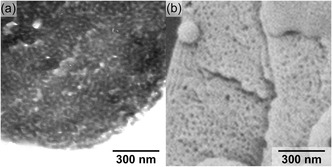
Analysis of inner structure. a) TEM cross section image of a P4VP cubosome embedded in resin and stained with iodine. b) SEM image of a fractured cubosome.

The PCs with the highest quality were further characterized regarding inner structure with electron microscopy (**Figure** **4**). For transmission electron microscopy (TEM) characterization, the PCs were freeze‐dried, embedded in a resin, and cut in 70‐100 nm thick slices. After staining the slices with iodine to enhance the contrast of the P4VP wall, cross sections analyzed by TEM validated the bicontinuous structure and the porosity throughout the microparticles. However, due to their spherical shape, the mesoscopic lattice is polycrystalline with only short‐range order (Figure 4a).^[^ ^39^ ^]^ The SEM imaging of fractured PCs provides additional information about the porosity. For that, PCs were cracked open by carefully cooling a PC dispersion in water with liquid nitrogen, where cracks in the ice can likewise fracture the PCs. From Figure 4b, it can be seen that the lattice becomes more polycrystalline toward the core of this particle, while the surface shows a more ordered pore structure. We previously reported PCs with monocrystalline lattices and long‐range order,^[^ ^49^ ^]^ where the absence of grain boundaries correlated with a strongly facetted surface of the PCs, even leading to the formation of terraces. We attributed this order to the formation process and the chain packing. That we do not obtain such a high order here, might be ascribed to the rather soft nature of the plasticized P4VP. Despite the high glass transition temperature of P4VP (*T*_g_ = 147 °C),^[^ ^54^ ^]^ we found that PEO‐*b*‐P4VP PCs are rather soft during handling, likely due to plasticization by water.^[^ ^55^ ^]^ The spherical boundary of the droplet during PC formation imposes a confinement in form of the high energy barrier toward the surrounding solvent mixture with an ever increasing water content. Given a high enough curvature and interfacial energy, the PCs will have to adapt to the curved surface resulting in a conflict of polyhedral lattice symmetry and droplet curvature. This conflict is resolved by the formation of grain boundaries, lattice defects, and ultimately, a polycrystalline inner structure with only short‐range order. Despite that, the entire inner structure of these particles consists of a bicontinuous network of interpenetrating channels, which is one of the most important requirements for mass transport into and distribution throughout the microparticles.

### 2.3 Coordination of P4VP Sites in the Cubosome Wall

**Figure 5:**
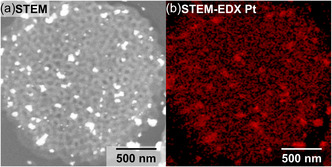
Pt‐loading of the P4VP cubosomes. a) STEM and b) STEM–EDX (Pt) measurements on cross‐sections of a PEO‐*b*‐P4VP cubosome loaded with Pt and embedded in resin.

We next investigated whether the inherent coordinative sites within the cubosome wall still remained addressable, e.g., by introducing transition metal salts and organic dyes through the solvent‐filled channels. Note that this is a different process than loading the PC channel system itself, which usually relies on supramolecular interactions between cargo and the PEO corona. To demonstrate the loading capability of P4VP, K_2_PtCl_4_ was added to a freshly prepared PC dispersion (molar ratio Pt/4VP 1:1) and stirred for 8 h at room temperature (rt). Subsequently, the Pt^2+^ was reduced to Pt^0^ nanoparticles with 3 eq. ascorbic acid at rt overnight. The Pt‐loaded samples were embedded in a resin, cut on an ultramicrotome, and analyzed by scanning TEM (STEM) with energy‐dispersive X‐ray spectroscopy (EDX, **Figure** **5**). In high‐angle annular dark‐field STEM, the intensity is proportional to the atomic number Z^1.6^. Therefore, regions with enriched Pt concentration give rise to bright contrast. The PC morphology was successfully preserved during loading and reduction, and Pt nanoparticles can be detected throughout the entire lattice of the cross section, as can be seen in Figure 5a. There are larger bright chunks of Pt that likely originate from a slight excess of K_2_PtCl_4_ that was not washed out before reduction. The element specific detection in Figure 5b likewise shows Pt coinciding with the location of the P4VP walls. The Pt^2+^ ions must have served as coordinative crosslinking sites within the P4VP wall, as ascorbic acid (≈20 mmol) would otherwise dissolve the PCs by lowering the pH of the aqueous solution below 4.

**Figure 6:**
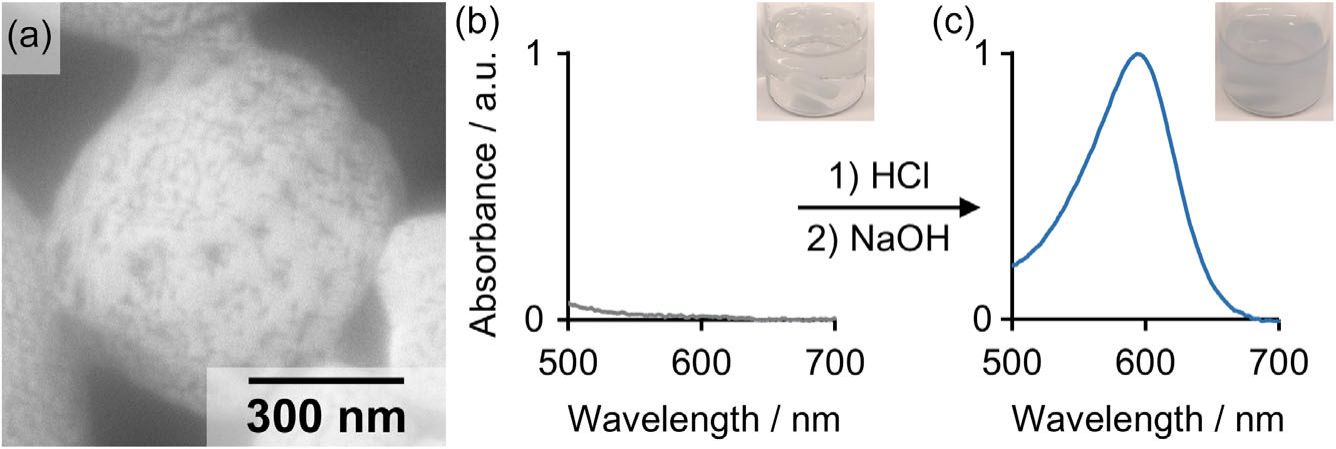
Dye‐loading of the P4VP cubosomes. a) SEM image of a PC loaded with thymolphthalein. b) UV/vis spectrum and photo of the dye‐loaded PCs in basic solution and c) UV/vis spectrum and photo after release of thymolphthalein.

As a second example, we place organic molecules into the P4VP wall through supramolecular interactions (**Figure** **6**). In general, many organic substances could be loaded into the P4VP domain,^[^ ^56^, ^57^, ^58^ ^]^ but we employ thymolphthalein as a model dye, because it has a well‐known pH‐dependent emission (blue in basic conditions, p*K*_a_ = 9.9)^[^ ^59^ ^]^ and can be easily seen by eye (and quantified by ultraviolet–visible [UV–vis]). The phenolic hydroxyl groups of thymolphthalein serve as H‐bond donors and form strong interactions with 4VP units that act as H‐bond acceptors.^[^ ^60^, ^61^ ^]^ The dye was simply added to the BCP solution before self‐assembly and migrated into the PC wall during nanoprecipitation. Figure 6a shows that PCs can be synthesized successfully despite the presence of the thymolphthalein, although the overall quality decreased, and the surface appears partially closed. After the assembly, the PCs were washed with diluted NaOH to rinse off excess thymolphthalein until the basic dispersion was completely colorless (Figure 6b). Any remaining thymolphthalein should now be located inside of the PC wall and held by H‐bonding. By adding HCl to reduce the pH < 4, the PC dissolved entirely thereby releasing the thymolphthalein, which can be visualized by again changing the pH to > 10 with NaOH (Figure 6c). Although this experiment is intended to demonstrate the loading capabilities of this system, it is not limited to thymolphthalein or the very similar phenolphthalein, which worked analogously. Various other organic substances could be loaded and precisely released as well by carefully adjusting the pH.^[^ ^62^, ^63^ ^]^ For instance, the pH of the human skin has a pH value in this specific regime and loaded PCs could be embedded in gel formulations or wound dressings for topical medication.^[^ ^64^ ^]^

## 3 Conclusion

Functional PCs were self‐assembly from PEO‐*b*‐P4VP BCPs via nanoprecipitation in mixtures of DMF and sodium hydrogen carbonate (10 mM). The BCPs were synthesized with RAFT polymerization thereby reaching high P4VP block lengths of over 400 units to enter the range of hydrophobic block fractions required for cubosome formation. An optimization of the self‐assembly procedure was conducted, whereas the best results were obtained starting with 10 mg mL^−1^ BCP in DMF and an addition of 2 mL of 10 mM NaHCO_3_ solution at a rate of 0.5 mL h^−1^. Screening these parameters provided a better understanding of the self‐assembly mechanism for the P4VP system. The PC morphology could be verified by SEM und TEM measurements and the functionality of the 4VP units by loading examples involving platinum complexes and organic dyes. Ongoing works concern more elaborate studies on the performance of catalyst‐modified PCs in heterogenous transition metal and organocatalysis.

## 4 Experimental Section

4.1

4.1.1

##### Materials

All materials were used as received unless otherwise specified. PEO methyl ether (*M*_n_ ≈ 2000 g mol^−1^), DCC (99%), DMAP (>99%), 4‐cyano‐4‐(((dodecylthio)carbonothioyl)thio)pentanoic acid (RAFT agent, 97%), AIBN (98%), CDCl_3_ (99.8%), ammonia (aqueous solution, 28–30%), DMAE (99.5%), dimethyl(1,5‐dicyclooctadiene) platinum (Pt(cod)Me_2_, 97%), phenolphthalein, and thymolphthalein were purchased from Sigma‐Adrich. The 4VP (>95%) and 2VP (>95%) were supplied by Alfa Aesar and passed over a filter column of alumina right before the reaction. Potassium platinum (II) tetrachloride (99.9%), DMF (99%), TEA (99%), and sodium hydrogen carbonate (>99%) were purchased from Fisher. Potassium hydrogen carbonate was obtained from Riedel‐de‐Haen, the borate buffer pH 10 from Bernd Kraft, and ascorbic acid from AppliChem and toluene (>99%) from Acros. DCM was used from a solvent drying system. Diethylether had a technical grade. Water was obtained from a water purification system (Milli‐Q). The dialysis tubes had a molecular weight cutoff of 3500 g mol^−1^ and were soaked in water for at least 1 h prior use.

##### Instruments: SEC

SEC measurements were performed on a 1260 Infinity instrument (PSS, Mainz). The samples were prepared by dissolving 1 mg of polymer in 1 mL eluent. The sample was filtered using a 0.2 μm PTFE syringe filter prior injection. The SEC measurements for the mPEO and the PEO–CTA were carried out in tetrahydrofuran using SDV columns at a temperature of 40 °C and a poly(styrene) calibration. The measurements for polymers containing P4VP were carried out in DMF. The system was equipped with GRAM columns. The DMF contained 50 mM LiBr and was calibrated with a poly(styrene) standard as well.

##### Instruments: NMR

NMR measurements were carried out on a Bruker Avance II 400 and a Bruker NEO 400. The samples were dissolved in CDCl_3_ and calibrated using tetramethylsilan. Syringe pumps are instruments and should be separated from NMR. *Syringe pumps* of the type LA‐100 were used for all self‐assembly experiments.

##### Instruments: SEM

For SEM, samples were prepared by diluting them to the desired concentration and adding 10 μL on a clean silicon wafer. The liquid was evaporated under reduced pressure. The wafer was attached to a stub using conductive carbon tape. The samples were sputtered with gold (3–5 nm). The measurement was carried out on a Zeiss CrossBeam 340 dual‐beam microscope. For EDX measurements, SEM was carried out utilizing a probe‐side Cs‐corrected JEOL JEM 2200 fs operated at 200 kV equipped with an Oxford Instruments X‐Max 100TLE EDX detector.

##### Instruments: Ultramicrotomy

The nanostructures were freeze dried and embedded in a UV curing resin. Subsequently, cross sections of 70 nm thickness were cut using a Ultracut E microtome (Reichert/Leica). The films were transferred to a TEM grid for measuring.

##### Instruments: TEM

Cross sections of the particles were stained with iodine for 2 h and analyzed on a TEM (Libra 120, Carl Zeiss) with an acceleration voltage of 80 kV. A charge‐coupled device camera (Tröndle) was used to take the images.

##### Instruments: UV–vis Spectroscopy

UV–vis spectroscopy measurements were performed on a JASCO V‐750. The spectra were recorded with a resolution of 0.5 nm and a scan rate of 200 nm min^−1^. A standard quartz cuvette with a pathway of 1 cm was used. The system was calibrated with diluted NaOH solution, in which the spectra were recorded. The samples were prepared by diluting them 1:4 with the NaOH solution and passing them over a 0.45 μm nylon syringe filter.

##### Methods: Synthesis of the PEO–CTA

The PEO–CTA was synthesized by dissolving 1 g of PEO (*M*_n_ ≈  2000 g mol^−1^, 0.5 mol), 404 mg RAFT agent (1 mmol), and 12.2 mg DMAP (0.1 mmol) in 10 mL dry DCM within a 50 mL round bottom flask. In a separate flask, 516 mg DCC (2.5 mmol) were dissolved in 10 mL dry DCM. Both mixtures were stirred at 0 °C and purged with argon. Subsequently, the DCC solution was dropwise added to the reaction mixture in the 50 mL round bottom flask using a degassed syringe. The reaction mixture was further stirred for 2 h at 0 °C and overnight at rt. After the reaction, the solution was filtered, and the residue was washed with DCM. The filtrate was concentrated under reduced pressure and precipitated tree times in −20 °C cold diethyl ether. The product was dried under reduced pressure to give 733 mg of a yellow solid (65.6% yield, 99.5% functionalization [NMR], *Đ* = 1.02 [SEC]).

##### Methods: Synthesis of PEO‐b‐P4VP

In a typical reaction, 210 mg PEO–CTA (88.0 μmol) and 16.0 mL 4VP (150 mmol) were dissolved in 16 mL DMF and purged with argon for 30 min. To that, 5.05 mg AIBN (30.8 μmol) was added from a DMF stock solution. The reaction mixture was stirred at 80 °C and quenched in liquid nitrogen after a reaction time of 3, 4, 5, and 7 h for the four different polymers, respectively. The polymer was precipitated in diethyl ether three times and subsequently dried under reduced pressure. An orange solid was obtained (*M*_n,NMR_ = 22.0 − 47.1 g mol^−1^, *M*_n, SEC_ = 12.6 − 25.7 kg mol^−1^, *Đ* = 1.36 − 1.50).

##### Methods: Self‐Assembly of PEO‐b‐P4VP

In a typical experiment, 0.1–25 mg BCP was dissolved in 1 mL DMF in a 10 mL vial with a 1 × 10 mm stir bar and sealed with a septum. The solution was gently stirred during the self‐assembly. Depending on the experiment, 1–4 mL selective solvent was added within 1–4 h using a syringe pump. The needle was thereby submerged in the assembly solution. Water as well as different basic aqueous solutions (mostly 10 mM NaHCO_3_) were tested as nonsolvent. After the nanoprecipitation, the solutions were transferred into a dialysis tube (molecular weight cutoff 3500 g mol^−1^) and dialyzed against the nonsolvent with at least five bath changes and with at least 4 h in between. For other self‐assembly conditions, everything was kept the same, except either the BCP concentration was varied from 0.1–25 mg, nonsolvent volume was varied from 1–4 mL.

##### Methods: Loading with Platinum

For loading with Pt, 2 mL of a freshly prepared dispersion of PEO‐*b*‐P4VP cubosomes in 10 mM NaHCO_3_ was mixed with 5.5 mg K_2_PtCl_4_ (ratio Pt/4VP 1:1). After stirring at rt for 8 h, 7.0 mg ascorbic acid (3 eq.) was added. The reaction mixture was further stirred overnight at rt. The sample was purified *via* centrifugation and dried under reduced pressure. A dark solid was obtained.

##### Methods: Loading with Model Dyes

For the loading of model dyes, 0.5 mL of BCP3 solution in DMF (10 mg mL^−1^) and 0.5 mL of a pH indicator solution (0.1 g L^−1^) were placed within a 10 mL vial. The same self‐assembly procedure as described earlier was applied.

##### Statistical Analysis

For all determinations of pore diameter and pore spacing, 50 values were determined each using grayscale analysis. Their mean values and standard deviation are given respectively. ImageJ (v.154f) was used for this purpose.

## Conflict of Interest

The authors declare no conflict of interest.

## Author Contributions

**Marcel Schumacher**: Investigation (lead); Validation (lead); Visualization (lead); Writing—original draft (lead); Writing—review and editing (supporting). **Nadine Tänzer**: Investigation (supporting); Validation (supporting); Writing—original draft (supporting); Writing—review and editing (supporting). **Marius G. Braun**: Investigation (supporting); Validation (supporting); Writing—original draft (supporting); Writing—review and editing (supporting). **Manuel Trömer**: Investigation (supporting); Visualization (supporting); Writing—original draft (supporting). **Giada Quintieri**: Investigation (supporting); Supervision (supporting); Writing—original draft (supporting). **Mahima Goel**: Validation (supporting); Writing—review and editing (supporting). **Markus Heidelmann**: Investigation (supporting); Visualization (supporting); Writing—review and editing (supporting). **André H. Gröschel**: Conceptualization (lead); Funding acquisition (lead); Project administration (lead); Supervision (lead); Writing—original draft (supporting); Writing—review and editing (lead).

## Supporting information

Supplementary Material

## Data Availability

The data that support the findings of this study are available from the corresponding author upon reasonable request.
