# Supplementary material for: Poly(4‐Vinylpyridine)‐Based Cubosomes: Synthesis, Assembly, and Loading Capabilities
Source: Small Sci. 2024 Oct 17;4(12):2400274. doi: 10.1002/smsc.202400274 (PMC11935103; doi:10.1002/smsc.202400274)
Supplement: Supplementary file 1 — Supplementary Material [file SMSC-4-2400274-s001.pdf]

Supporting Information

to

Poly(4-Vinylpyridine)-Based Cubosomes: Synthesis, Assembly, and Loading Capabilities

Marcel Schumacher, Nadine Tänzer, Marius G. Braun, Manuel Trömer, Giada Quintieri, Mahima Goel, Markus Heidelmann, and André H. Gröschel

Supporting Figures

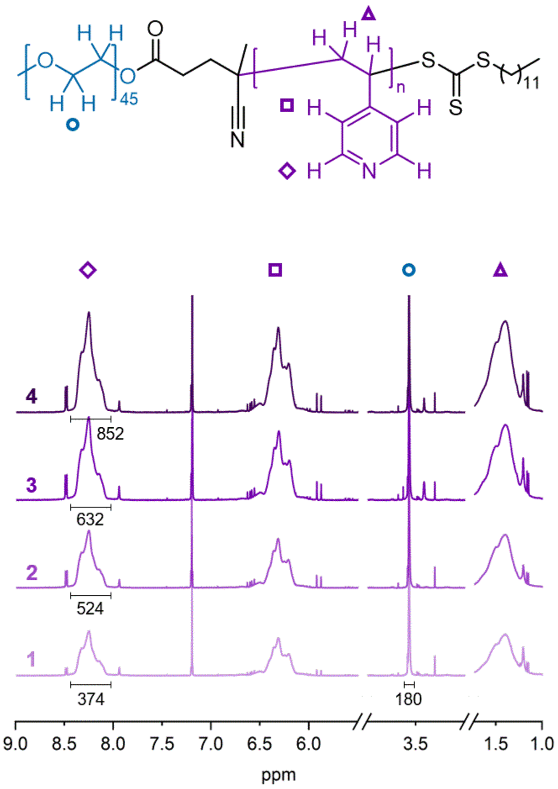

Figure S1. NMR spectra and integrals for the PEO-*b*-P4VP BCPs.

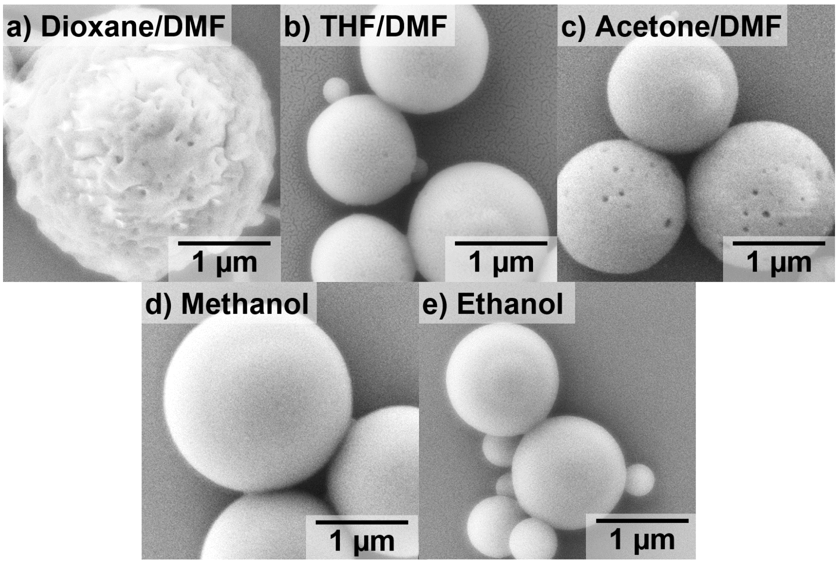

Figure S2. SEM images demonstration the influence of different common solvents on the self-assembly.

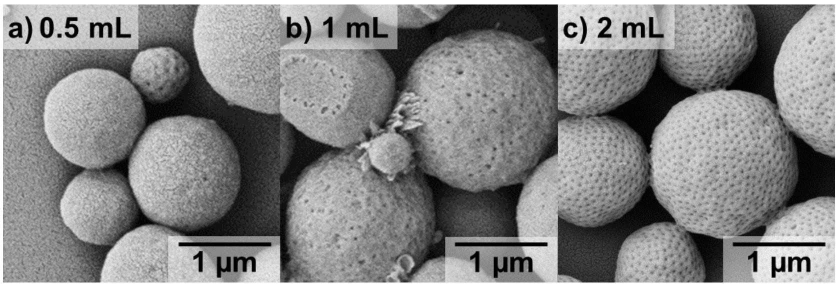

Figure S3. SEM images showing the influence of the addition amount of the 10 mM sodium hydrogen carbonate solution to 10 mg of polymer 3 (f_{P4VP} = 94.4%) in 1 mL DMF.

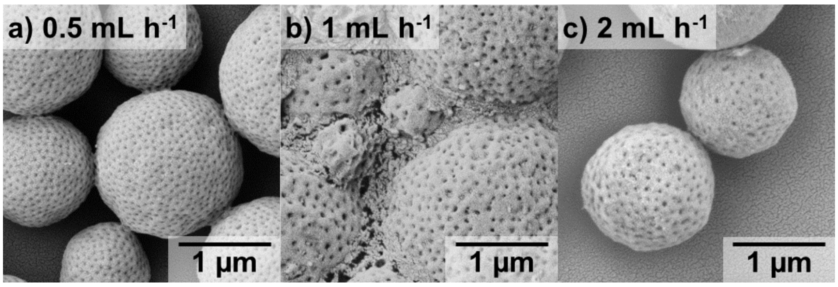

Figure S4. SEM images demonstrating the influence of the addition rate of the 2 mL 10 mM sodium hydrogen carbonate solution to 10 mg of polymer 3 (f_{P4VP} = 94.4%) in 1 mL DMF.
